# Supercritical Antisolvent Precipitation of Amorphous Copper–Zinc Georgeite and Acetate Precursors for the Preparation of Ambient‐Pressure Water‐Gas‐Shift Copper/Zinc Oxide Catalysts

**DOI:** 10.1002/cctc.201601603

**Published:** 2017-04-07

**Authors:** Paul J. Smith, Simon A. Kondrat, James H. Carter, Philip A. Chater, Jonathan K. Bartley, Stuart H. Taylor, Michael S. Spencer, Graham J. Hutchings

**Affiliations:** ^1^ Cardiff Catalysis Institute, School of ChemistryCardiff UniversityMain Building, Park PlaceCardiffCF10 3ATUK; ^2^ Diamond Light SourceDidcotOX11 0DEUK

**Keywords:** copper, gas-phase reactions, supercritical fluids, water, zinc

## Abstract

A series of copper–zinc acetate and zincian georgeite precursors have been produced by supercritical CO_2_ antisolvent (SAS) precipitation as precursors to Cu/ZnO catalysts for the water gas shift (WGS) reaction. The amorphous materials were prepared by varying the water/ethanol volumetric ratio in the initial metal acetate solutions. Water addition promoted georgeite formation at the expense of mixed metal acetates, which are formed in the absence of the water co‐solvent. Optimum SAS precipitation occurs without water to give high surface areas, whereas high water content gives inferior surface areas and copper–zinc segregation. Calcination of the acetates is exothermic, producing a mixture of metal oxides with high crystallinity. However, thermal decomposition of zincian georgeite resulted in highly dispersed CuO and ZnO crystallites with poor structural order. The georgeite‐derived catalysts give superior WGS performance to the acetate‐derived catalysts, which is attributed to enhanced copper–zinc interactions that originate from the precursor.

## Introduction

The water‐gas‐shift (WGS) reaction (CO+H_2_O⇌H_2_+CO_2_) is of central importance for the industrial production of hydrogen and depletion of CO in syngas streams.^1^, ^2^ Consequently, the WGS process is intimately linked with ammonia synthesis and hydrogen polymer electrolyte membrane fuel cell (PEMFC) applications for which CO is a poison. As the WGS process is an exothermic reversible reaction, the forward reaction is limited at higher temperatures. However, reaction rates are reduced at lower temperatures. To overcome this conflict the WGS process is performed in two stages, involving both a high‐temperature (310–450 °C) and low‐temperature (210–240 °C) step. The focus of this work is on the low‐temperature WGS process in which Cu/ZnO catalysts are employed, as they exhibit high activity under these conditions. Similar catalysts are also used to convert syngas to methanol where the WGS process is also relevant as a parallel reaction.^3^

Cu/ZnO catalysts have attracted a significant amount of industrial and academic interest because of their commercial importance. Experimental and theoretical studies have investigated the role of the individual catalyst components and the nature of the active sites. For methanol synthesis, key structural parameters for an optimised catalyst include an efficiently strong metal–support interaction (SMSI) and a large copper surface area that is highly defective with kinks, steps and faults.^4^, ^5^, ^6^ However, fewer studies have been conducted on the WGS reaction indicating that it is a far less understood process. Cu/ZnO catalysts are structurally dynamic and highly sensitive to the reaction conditions and therefore are expected to behave differently in these chemical processes.^7^, ^8^, ^9^ Despite this, copper is still widely considered the active component of the catalyst whereas there have been conflicting reports on the role of ZnO.^10^, ^11^ Many research groups have reported that WGS activity is correlated to Cu surface area,^12^, ^13^, ^14^, ^15^, ^16^, ^17^ whereas several groups have indicated that there is no correlation.^10^, ^18^ Interestingly, Hadden and co‐workers reported that discrete Cu‐surface‐area–activity relationships only exist between groups of catalysts prepared similarly.^19^ Furthermore, although it is generally accepted that water dissociation is the rate‐limiting step,^10^, ^11^, ^20^, ^21^, ^22^ studies have reported either an associative^11^, ^18^ or regenerative^15^, ^22^, ^23^ mechanism is occurring, or even both.^1^, ^24^

The preparation of commercially used Cu/ZnO catalysts is well understood and has remained largely unchanged since its commercialisation in the early 1960s.^10^ Aqueous metal nitrate and sodium carbonate solutions are simultaneously added under controlled pH and temperature conditions to yield, after subsequent ageing, intimately mixed copper–zinc hydroxycarbonate catalyst precursors. Hydrotalcite has been reported as the optimum precursor for WGS catalysts^16^ that, in addition to copper and zinc, includes alumina. Owing to the high partial pressure of steam required for industrial operating conditions, the incorporation of alumina into the catalyst is essential to promote stability and hence catalyst performance.^1^, ^15^, ^18^ Typical compositions of commercial WGS catalysts are approximately 33 % CuO, 34 % ZnO and 33 % Al_2_O_3_. Interestingly, some co‐precipitation preparations have used a constant pH of 9^15^, ^25^, ^26^ because more strongly alkaline conditions are likely to favour hydrotalcite formation. However, titration studies performed by Behrens and co‐workers have identified that a constant pH of 7 is required for the simultaneous precipitation of all metal species and preparation of well‐mixed hydroxycarbonates.^3^, ^27^ Calcination of hydroxycarbonates under optimum conditions produces mixed metal oxides that retain residual carbonate, with final‐state catalysts produced by subsequent reduction of the CuO crystallites to Cu. Many authors correlate the performance of the final‐state catalyst to each of these individual catalyst synthesis steps and describe it as the chemical memory of the catalyst.^28^, ^29^ This can be problematic because the co‐precipitation process is highly sensitive to a wide range of parameters including pH, temperature and ageing, which all contribute to the microstructural properties of the resulting catalyst precursor.

We have recently prepared zincian georgeite, an amorphous copper–zinc hydroxycarbonate, by supercritical CO_2_ antisolvent (SAS) precipitation and have demonstrated that it can be used to prepare Cu/ZnO catalysts that are highly active and stable for the WGS reaction.^30^ This enhanced stability removes the necessity to incorporate alumina into the catalyst. Furthermore, this procedure enables the processing of high‐purity materials devoid of residual catalyst poisons, including nitrates^28^ and alkali metals,^31^ and does not require delicate control of a broad range of conditions such as pH. Formation of zincian georgeite in the SAS process occurs when copper‐zinc acetate solutions, prepared by using an ethanol–water solvent, come into contact with supercritical CO_2_ (scCO_2_) to induce nucleation and precipitation. It was found that in the absence of water, a mixed copper–zinc acetate was predominantly prepared instead. We have observed similar findings in related SAS preparations including the synthesis of cobalt zinc oxide catalysts for the Fischer–Tropsch reaction,^32^ transition metal oxides for propane oxidation^33^ and mixed copper–manganese phases for CO oxidation.^34^, ^35^ In each case, in situ formation of carbonic acid occurs from water and scCO_2_ that subsequently dissociates to CO_3_^2−^ anions for carbonate formation. In this work, we performed a thorough investigation on the effect of the water content on the microstructural properties of the resulting SAS prepared materials using 0–30 vol. % H_2_O/EtOH solutions. This work allows for a better understanding of the SAS process in addition to optimising the preparation of Cu/ZnO catalysts. Copper–zinc acetate and zincian georgeite phases were examined as precursors for Cu/ZnO catalysts and tested for the WGS reaction at ambient pressure. The catalyst performances could then be correlated to their structures, which highlighted the importance of the catalyst precursor phase.

## Results and Discussion

### SAS‐prepared catalyst precursors

**Figure 1:**
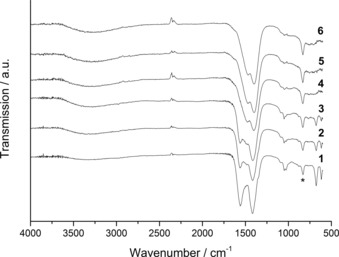
FTIR spectra of the SAS‐prepared materials formed by using different solvent mixtures. (1) 0 vol. % H_2_O/EtOH, (2) 0.5 vol. % H_2_O/EtOH, (3) 1 vol. % H_2_O/EtOH, (4) 5 vol. % H_2_O/EtOH, (5) 10 vol. % H_2_O/EtOH and (6) 30 vol. % H_2_O/EtOH. *Carbonate *ν*_2_ band (835 cm^−1^) marked for clarity.

**Figure 2:**
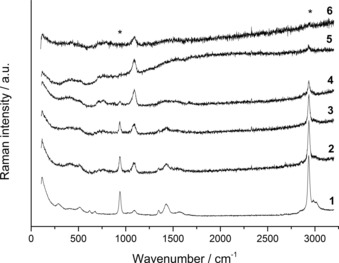
Raman spectra of the SAS‐prepared materials formed using different solvent mixtures. (1) 0 vol. % H_2_O/EtOH, (2) 0.5 vol. % H_2_O/EtOH, (3) 1 vol. % H_2_O/EtOH, (4) 5 vol. % H_2_O/EtOH, (5) 10 vol. % H_2_O/EtOH, and (6) 30 vol. % H_2_O/EtOH. *Acetate bands (938 and 2934 cm^−1^) that remain at higher water contents are marked for clarity.

Initially, vibrational spectroscopy techniques were used to identify the phases produced by using various water contents, as well as to determine changes in the molecular structure of the SAS precipitates. FTIR spectroscopy confirmed the predominant phase to be a metal acetate if no water co‐solvent was used, and zincian georgeite if ≥5 vol. % water co‐solvent was utilised (Figure 1). Interestingly, materials prepared by using 0.5–1 % water clearly show a phase mixture of acetate and zincian georgeite, as bands for both phases are apparent. FTIR analysis was also conducted on the starting materials for comparison (Figure S1, Supporting Information), with a complete assignment of bands for all materials conducted in accordance with the literature (Table 1).^36^, ^37^, ^38^ The SAS‐prepared materials have fewer bands and are broader. For zincian georgeite, carbonate bands are present at 1475, 1404, 1048 and 835 cm^−1^ with a broad O−H stretch centred at 3289 cm^−1^. The metal acetate, in comparison, displays bands at 1561 and 1420 cm^−1^, assigned to COO^−^ asymmetric and symmetric stretches, respectively, and bands at 1053 and 1043 cm^−1^ are assigned to *δ*(C−H) modes. Interestingly a broad O−H band is also present, albeit at lower intensity, as well as a carbonate band at 832 cm^−1^ even if no water was used. The trace amounts of zincian georgeite present are attributed to the small amount of water occluded in the hydrated starting metal acetate salts. Similar findings have been reported by Reverchon and co‐workers for the SAS precipitation of zinc acetate.^39^ Raman analysis also identified carbonate bands for the zincian georgeite materials at 1091, 771 and 724 cm^−1^ (Figure 2, Table 1). However, acetate bands were also identified at 2934 and 938 cm^−1^, assigned to *ν*(C−H) and *ν*(C−C) modes, respectively. These bands are weakly visible even if using water contents ≥10 vol. %, illustrating that the complete removal of acetate is challenging. We noticed though that a precipitate formed in our metal acetate solutions with water contents ≥5 vol. %, before SAS precipitation. Raman spectroscopy confirmed it was an acetate after comparison against the as‐received acetate reagents (Figure S2). This acetate present in our solutions would also be incorporated into our resulting precipitates. The wavenumber for the *ν*(C−C) mode has also been correlated by Quiles and Burneau to the acetate coordination mode in copper complexes.^37^ This wavenumber was 949 cm^−1^ for the as‐received copper acetate monohydrate salt and 938 cm^−1^ for all the SAS prepared materials, which strongly matches bidentate and pseudo‐bridging modes, respectively. This suggests a distortion in the acetate ligand after SAS preparation, possibly attributed to OH^−^ anions that were also incorporated into the structure.

**Table 1:** Wavenumbers [cm^−1^] and assignments of the vibrational modes of copper(II) acetate monohydrate, zinc(II) acetate dihydrate and selected SAS prepared materials between 4000 and 400 cm^−1^.

Copper(II) acetate		Zinc(II) acetate		SAS prepared copper‐zinc		SAS prepared zincian		Band
monohydrate		dihydrate		acetate^[a]^		georgeite^[b]^		assignment
FTIR	Raman	FTIR	Raman	FTIR	Raman	FTIR	Raman	
3489, 3367, 3304	3022	3378, 3141	3059	3275	3496	3289	3381	*ν*(O−H)
2989, 2940	3004, 2942		3031, 2938		2934		2934	*ν*(C−H)
1603, 1597		1560		1560	1566			*ν*_a_(COO)^−^
1444, 1418	1418	1445	1455	1420	1428			*ν*_s_(COO)^−^
						1475, 1404		*ν*_3_(CO_3_)^2−^
1354	1441, 1362	1382	1361	1344	1347			*δ*(CH_3_)
1056, 1036		1051, 1024		1053, 1043				*γ*(CH_3_)
					1092	1048	1091	*ν*_1_(CO_3_)^2−^
	949	951	954		938		938	*ν*(C−C)
				832		831		*ν*_2_(CO_3_)^2−^
							771, 724	*ν*_4_(CO_3_)^2−^
696, 632	703	701, 619	693, 644	681, 619	677, 612			*δ*(COO)^−^
			478		503			*γ*(COO)^−^

Notations are used for stretching (*ν*), bending (*δ*) and rocking modes (*γ*). [a] Prepared using 0 vol. % H_2_O/EtOH. [b] Prepared using 10 vol. % H_2_O/EtOH.

**Figure 3:**
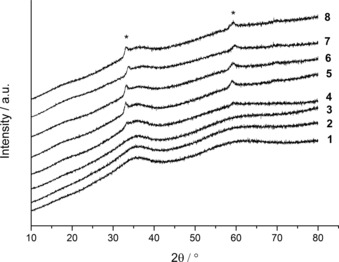
XRD patterns of the SAS prepared materials formed by using different solvent mixtures. (1) 0 vol. % H_2_O/EtOH, (2) 0.5 vol. % H_2_O/EtOH, (3) 1 vol. % H_2_O/EtOH, (4) 5 vol. % H_2_O/EtOH, (5) 10 vol. % H_2_O/EtOH, (6) 15 vol. % H_2_O/EtOH, (7) 20 vol. % H_2_O/EtOH and (8) 30 vol. % H_2_O/EtOH. *Reflections (33.1 and 59.1°) associated with acetate precipitate obtained from the starting metal acetate solution.

**Figure 4:**
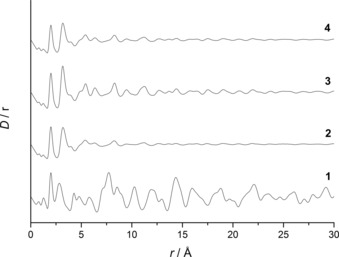
Observed PDF D(*r*) data for (1) as received copper acetate and SAS prepared materials formed by using different solvent mixtures. (2) 10 vol. % H_2_O/EtOH (3) 15 vol. % H_2_O/EtOH and (4) 20 vol. % H_2_O/EtOH. The absence of a well pronounced peak at 15.27 Å in the SAS prepared materials indicates zincian malachite is not present.

From XRD analysis it was concluded that all the SAS‐prepared materials are amorphous regardless of water content (Figure 3), in comparison to the high level of crystallinity displayed for the starting materials (Figure S3 a). However, two small reflections at 33.1 and 59.1° are present from using water contents ≥5 vol. %, and these matched the reflections of the acetate precipitate formed in the metal acetate solution. A comparison with the XRD pattern of our zincian georgeite materials against this acetate determined that it is only a minor component of the sample (Figure S3 b). The structural features of the zincian georgeite prepared with water contents ≥10 vol. % were investigated further by PDF and compared against the as‐received copper acetate monohydrate as a standard (Figure 4). The zincian georgeite phase remains largely unchanged regardless of the amount of water used, although an extended order is evident for water contents ≥15 vol. %. It also cannot be ruled out that some structural contributions originate from the acetate impurity. The lack of a distinct fingerprint band for malachite at 15.27 Å, which correlates to crystallography identical copper atoms in adjacent unit cells, also indicates no evidence of ageing. In contrast, the as‐received copper acetate monohydrate displays a high level of order as determined by XRD analysis (Figure S3 a). Similarities with the zincian georgeite samples in the local order end at distances <2.5 Å. We have previously shown that the metal cations in georgeite adopts a distorted octahedral environment^30^ whereas copper acetate monohydrate exists as a dinuclear structure. Both phases give metal–carbon and metal–oxygen distances of 1.23 and 2 Å, respectively, with the lack of a shoulder on the latter suggesting Jahn–Teller contributions are not significant in both cases. A metal–metal distance of 2.82 Å for copper acetate monohydrate and 3.19 Å for zincian georgeite highlights clear differences in the local structures.

**Figure 5:**
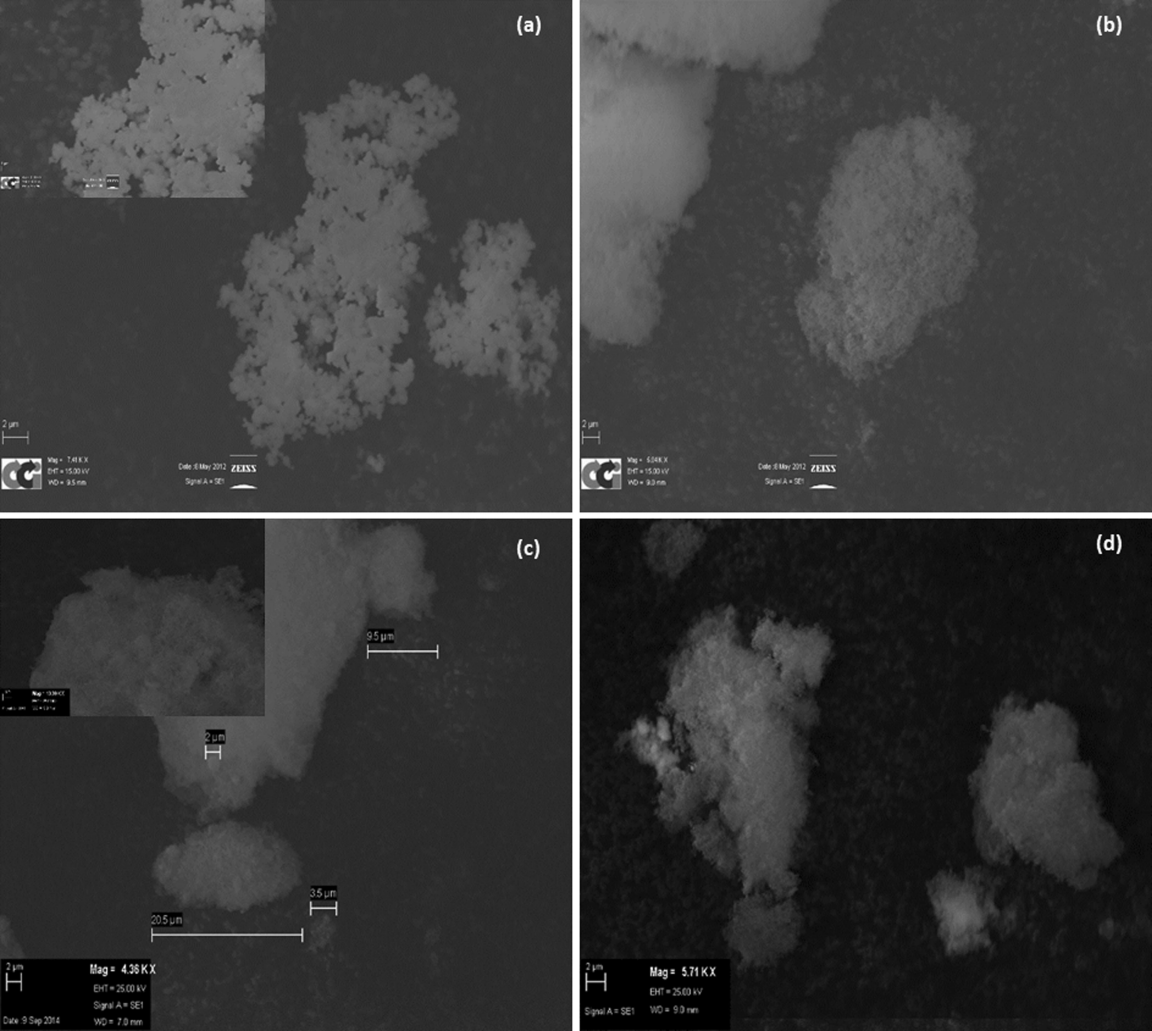
SEM images of (a) 0 vol. % H_2_O/EtOH, (b) 1 vol. % H_2_O/EtOH, (c) 10 vol. % H_2_O/EtOH and (d) 30 vol. % H_2_O/EtOH precursors. Insets in (a) and (c) are images taken at higher magnification to emphasise differences in morphology.

Scanning electron microscopy (SEM) was used to identify further differences between the SAS‐prepared materials. The morphology of the acetate prepared without water was found to consist of defined, spherical agglomerations of fibrous strings (Figure 5 a). The cluster sizes varied significantly depending on the degree of agglomeration. In comparison, the precursors prepared with water all display a very different morphology (Figures 5 b–d). Although agglomeration was still apparent, the resulting particles formed cloud‐like morphologies that lacked defined edges. We noted similar observations during SAS preparations of copper manganese oxides and concluded that water addition to the SAS process facilitates a change in precipitation mechanism.^35^ This mechanistic change is governed by the introduction of surface tension into the system, resulting from the lack of miscibility between water and scCO_2_. Consequently, a diffusion‐based mechanism would dominate causing scCO_2_ to diffuse into the solution droplets instead of instant gas‐like mixing of these phases.

We next quantified the metal compositions of the precipitates and calculated their yields from their SAS effluents by energy‐dispersive X‐ray (EDX) and microwave‐plasma atomic emission spectroscopy (MP–AES) analysis (Table 2). The water content had a significant effect, with a clear trend apparent that divided these materials into three groups. Without water addition, the nominal Cu/Zn molar ratio of 2:1 is not achieved with a higher zinc content giving a 1.4:1 ratio. This is attributed to the partial solubility of the acetate precipitate in the EtOH/scCO_2_ phase, with preferential leaching of copper over time during the synthesis. To overcome this, the antisolvent strength of the scCO_2_ would need to be increased by increasing the scCO_2_/solvent ratio to reduce the acetates affinity with the EtOH solvent. However, using just 0.5 vol. % water achieved the desired Cu/Zn molar ratio with 100 % metal yields. This shows that copper is less soluble in the resulting H_2_O/EtOH/scCO_2_ phase, as there is no more leaching. This is apparent up to water contents ≥15 vol. %, at which the Cu/Zn ratio increases above 2:1 and significant levels of both metals are present in the effluent. In this case, incomplete metal precipitation is attributed to the formation of a biphasic system during the precipitation process. The high zinc content in these effluents suggests that it has a higher affinity with water than copper. As noted previously in the preparation of CuMn_2_O_4_, there is an optimal water concentration required to produce carbonate precipitates of the correct composition and morphology.^35^ Very low water contents in the synthesis mixture result in significant retention of the acetate counterion whereas too great content results in breakdown of the supercritical phase system.

**Table 2:** EDX and MP–AES analysis of the SAS‐prepared materials and their effluents formed by using various solvent mixtures.

Water content	Atomic		Cu/Zn	Effluent concentration		Yield [%]		
[%]	[%]^[a]^		molar	[mg L^−1^]^[b]^				
	Cu	Zn	ratio	Cu	Zn	Cu	Zn	Total
0	58	42	1.4:1	264	8	79	99	89
0.5	67	33	2:1	0	0.1	100	100	100
1	67	33	2:1	0	0.2	100	100	100
5	67	33	2:1	0	0.3	100	100	100
10	67	33	2:1	0	0.5	100	100	100
15	68	32	2.1:1	0.1	0.8	100	100	100
20	71	29	2.4:1	16	68	99	89	94
30	75	25	3.1:1	128	332	90	48	69

[a] Standard deviation ±1 at. %. [b] Standard deviation ±0.08 mg L^−1^.

### Calcined materials

**Figure 6:**
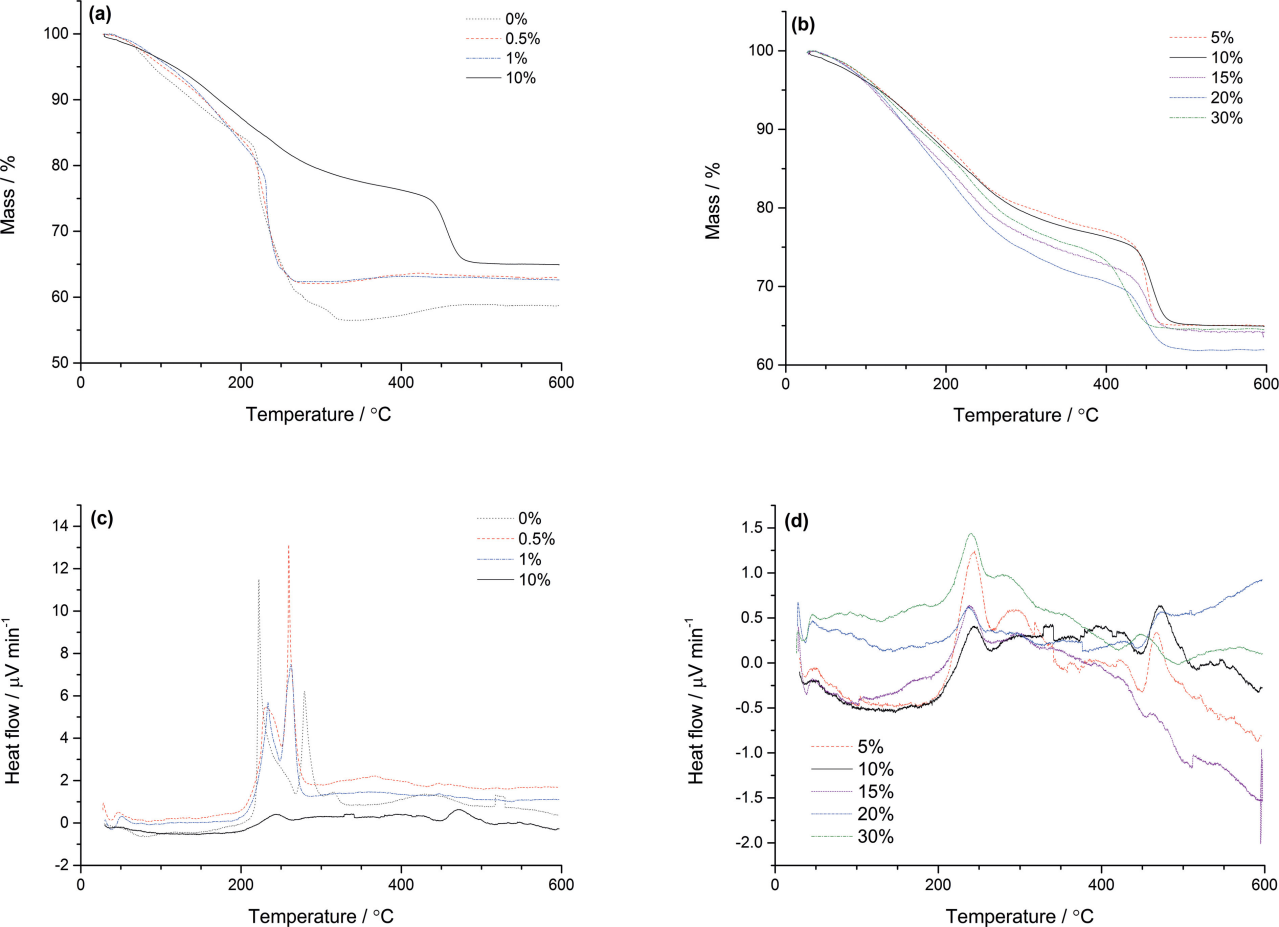
TGA/DTA of the SAS prepared materials formed by using various solvent mixtures, with water contents indicated as in the figure. (a) TGA of materials prepared by using 0–1 vol. % H_2_O/EtOH mixtures, with 10 vol. % H_2_O/EtOH included for clarity. (b) TGA of materials prepared by using 5–30 vol. % H_2_O/EtOH mixtures. (c) DTA of materials prepared by using 0–1 vol. % H_2_O/EtOH mixtures, with 10 vol. % H_2_O/EtOH included for clarity. (d) DTA of materials prepared by using 5–30 vol. % H_2_O/EtOH mixtures.

The thermal behaviour of the SAS precipitates was then studied by thermal gravimetric analysis/differential thermal analysis (TGA/DTA) to trial their applicability as suitable precursors for WGS catalysts (Figure 6). Decomposition profiles of these phases are known to be dependent on sample mass^40^, ^41^ so the amounts of samples analysed were kept constant. To understand the decomposition of the SAS precipitates, we must consider the thermal decomposition of the as‐received copper acetate monohydrate.^42^ The thermal decomposition of acetate salts is complex and can be highly exothermic. A phenomenon known as auto‐reduction can occur because CO is produced from the decomposition of acetate ligands during metal oxide formation. Easily reducible metal oxides such as copper oxide can reduce to the metallic state, and then, in a supply of oxygen, can gradually re‐oxidise and regain mass with temperature. Less reducible metal oxides, such as ZnO, remain unaffected. With regard to the SAS precipitated materials, the auto‐reduction process was apparent for samples prepared using 0–1 vol. % water, with the high exothermic decomposition characteristic of metal acetates (Figures 6 a,c, Table 3). Clearly, the acetate decomposition dominates the TGA profile even if discernible quantities of zincian georgeite are present. The mass gain from auto‐reduction of the acetates also correlates to the amount of acetate in the precipitate. Furthermore, the final mass loss of the sample prepared without water was approximately 5 % lower and had a different DTA profile in comparison with the acetate/zincian georgeite biphasic samples. This suggests that the presence of zincian georgeite still influences the decomposition in these acetate‐rich materials. Precursors prepared with water contents ≥5 vol. % had TGA/DTA profiles that are characteristic of zincian georgeite (Figures 6 b,d).^30^ The thermal process occurs in three steps, with loss of occluded water at temperatures ≤100 °C, simultaneous loss of hydroxyl and carbonate groups up to 300 °C and loss of high‐temperature carbonate (HT‐CO_3_) species seen at temperatures >420 °C. All these materials had a final mass loss of 33–36 %, leaving 13–15 % residual mass after reaching 300 °C at which HT‐CO_3_ is still retained. Interestingly, HT‐CO_3_ remains stable up to 430 °C for water contents 5–20 vol. % but begins to decompose at 400 °C if using 30 vol. % water. The temperature of HT‐CO_3_ decomposition may correlate with the degree of mixing between copper and zinc and hence the overall thermal stability of the material. As mentioned previously, using 30 vol. % water causes separation of the H_2_O/EtOH/scCO_2_ phase in the SAS system, resulting in poor metal mixing and segregation in the resulting precipitates.

**Table 3:** Effect of water content (0–1 vol. %) on observed TGA findings.

Water content	Sample mass	Mass loss from	Final mass	Mass gain
[%]	[mg]	decomposition [%]	loss [%]	[%]
0	11.9	43.5	41.1	2.4
0.5	13.5	38	36.4	1.6
1	11.6	37.7	36.8	0.9

**Figure 7:**
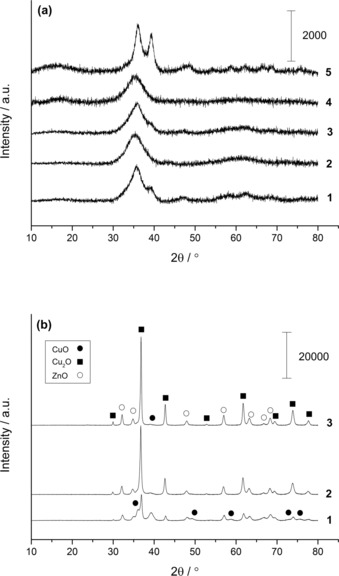
a) XRD patterns of calcined materials prepared by SAS precipitation using various solvent mixtures. (1) 5 vol. % H_2_O/EtOH, (2) 10 vol. % H_2_O/EtOH, (3) 15 vol. % H_2_O/EtOH, (4) 20 vol. % H_2_O/EtOH and (5) 30 vol. % H_2_O/EtOH. All ill‐defined reflections present are associated with CuO. b) XRD patterns of calcined materials prepared by SAS precipitation using various solvent mixtures. (1) 1 vol. % H_2_O/EtOH, (2) 0.5 vol. % H_2_O/EtOH and (3) 0 vol. % H_2_O/EtOH.

XRD analysis was performed on the materials after calcination at 300 °C to examine the structural changes (Figures 7 a,b). All the precursors were amorphous (Figure 3), but the precipitates prepared with water contents 0–1 vol. % were found to be highly crystalline after calcination. The reflections sharpened and increased in intensity to give larger crystallite sizes as the water content decreased (Table 4). Phase analysis determined that these materials are composed of CuO, Cu_2_O and ZnO. From semi‐quantitative analysis using relative intensity ratio analysis it was also concluded that the Cu^+^/Cu^2+^ ratio increased with decreasing water content, as expected from the enhanced auto‐reduction. Interestingly, no metallic Cu reflections were identified, which suggests that the copper had not fully reduced, or that it had partially re‐oxidised during thermal treatment. In contrast, the XRD patterns of the calcined precipitates prepared with water contents ≥5 vol. % showed poorly defined CuO reflections, suggesting the presence of nano‐crystalline CuO/ZnO phases. This can be attributed to the absence of a highly exothermic decomposition observed for the acetate materials (Figures 6 c,d). Samples prepared with 5 and 30 vol. % displayed more defined reflections. The former had a higher content of acetate present (Figure 2) resulting in a slightly higher exothermic decomposition, whereas the latter had poorer dispersion and mixing of copper and zinc.

**Table 4:** XRD crystallite sizes and semi‐quantitative analysis of Cu_2_O, CuO and ZnO phases after calcination of SAS prepared materials formed by using 0–1 vol. % H_2_O/EtOH mixtures.

Water content	Crystallite size [nm]			Phase composition [%]^[a]^		
[%]	Cu_2_O	CuO	ZnO	Cu_2_O	CuO	ZnO
0	26.6	6.5	14.4	77	10	13
0.5	19	–	11.9	70	12	17
1	19.5	–	10.9	35	37	28

[a] Calculated using relative intensity ratio analysis.

Surface areas of all the materials were measured before and after calcination by using the BET method (Table 5). Prior to calcination, the samples prepared with 0.5–1 vol. % water contents produced the highest surface areas in the range of 150–160 m^2^ g^−1^. Surprisingly, the sample prepared without water had a very low surface area, which could be attributed to copper leaching (Table 2) and its different morphology (Figure 5 a). These findings imply that optimum precipitation and facilitation of atomically mixed metal precipitates can only be achieved in the absence of water in the SAS process. Reverchon and co‐workers have reported that optimum nanoparticle formation can only occur if operating within a single, homogeneous system.^43^ To achieve this, it is essential to operate at pressures higher than the mixture critical point (MCP). The introduction of more components causes the MCP to shift to higher pressures. The poor miscibility of water in scCO_2_ is likely to significantly alter the MCP. Therefore, poorer metal dispersion, reduced surface area and larger particle formation are all expected with increasing water content at fixed pressure. The surface area decreases markedly between water contents of 1 and 5 vol. % but remains in the range of 90–100 m^2^ g^−1^ up to 20 vol. % water. It then decreases to 65 m^2^ g^−1^ for 30 vol. % water at which content the presence of scCO_2_‐rich and water‐rich phases are likely to co‐exist during the SAS precipitation. The BET surface areas for the calcined materials are in agreement with the XRD and DTA data (Figures 6 c,d, 7). The highly exothermic decomposition of the acetate phases resulted in highly crystalline materials with large crystallite sizes and low surface areas. With higher water contents, at which decomposition of the zincian georgeite phase dominates the overall decomposition, the surface areas only decreased slightly. These materials will still have residual carbonate remaining to suppress metal‐oxide crystallite growth, whilst the thermal decomposition process has gone through to completion for the copper‐zinc acetates.

**Table 5:** BET surface areas of SAS‐prepared materials formed by using various solvent mixtures before and after calcination. C values are provided to indicate the degree of accuracy of each analysis.

Water content	Precursor		Calcined	
[%]	surface area	*C* value	surface area	*C* value
	[m^2^ g^−1^]^[a]^		[m^2^ g^−1^]^[a]^	
0	17	22	19	443
0.5	162	30	20	104
1	152	29	27	281
5	87	65	67	367
10	97	79	85	89
15	99	85	76	368
20	104	129	77	298
30	65	75	48	472

[a] Standard deviation ±6 m^2^ g^−1^.

### Catalyst reduction and WGS testing

**Figure 8:**
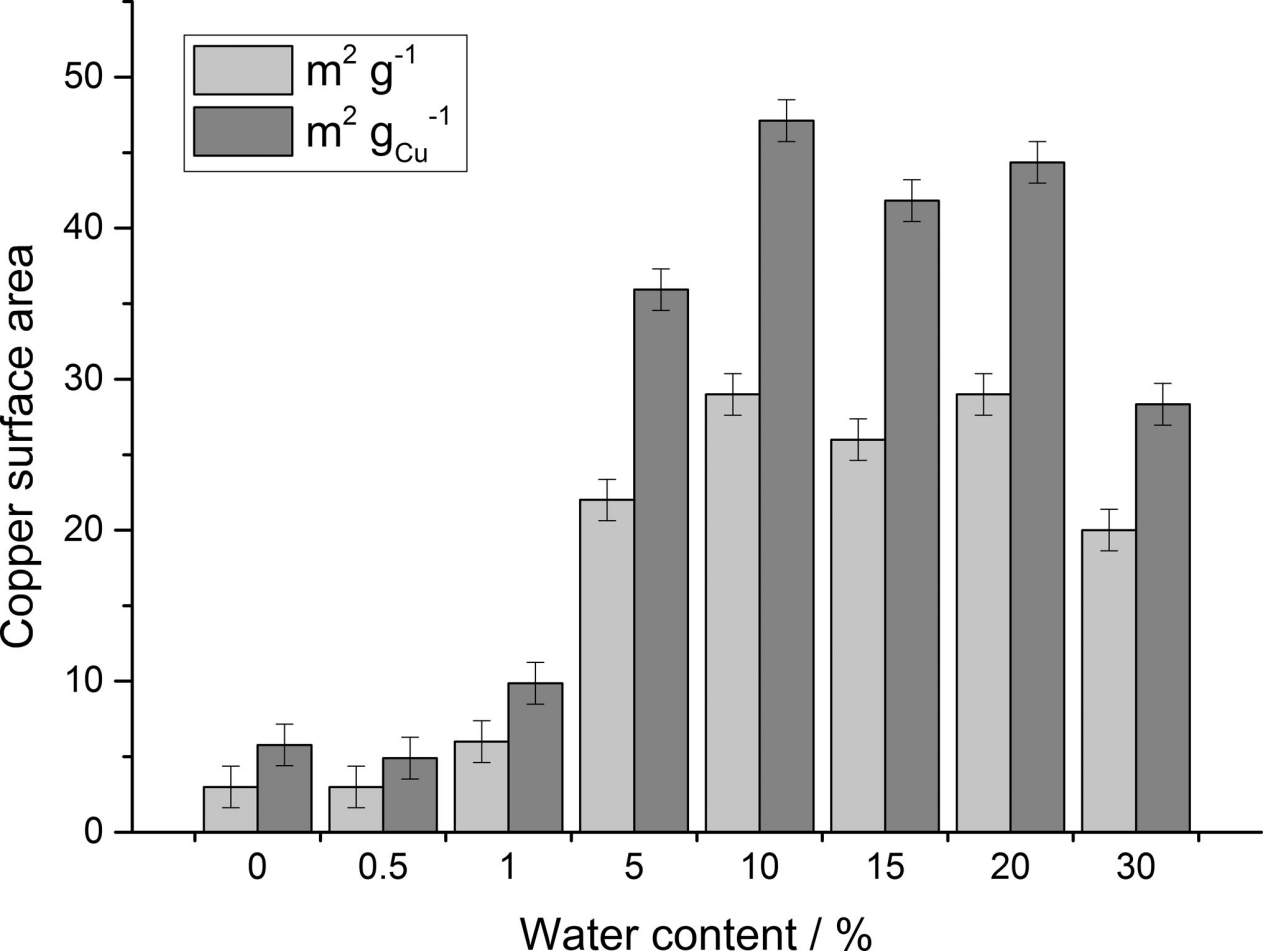
Copper surface area analysis (light grey) of Cu/ZnO catalysts prepared by SAS precipitation using various H_2_O/EtOH solvent mixtures, with water vol. % contents indicated. Copper surface areas normalised to copper mass (dark grey) are also provided and assume only Cu and ZnO phases present before analysis.

We performed copper surface area analysis of the final‐state catalysts for catalyst screening (Figure 8). As the EDX findings illustrated that the Cu/Zn molar ratios of the materials were not the same (Table 2), the values were also normalised to copper mass. The acetate‐derived catalysts prepared with 0–1 vol. % water displayed the lowest copper surface areas, with poor dispersion of the metallic copper attributed to phase segregation and large CuO_*x*_ crystallite formation after calcination. Catalysts prepared with water contents between 10 and 20 vol. % were found to have higher copper surface areas in the range of 26–29 m^2^ g^−1^. The slightly lower values apparent for the 5 and 30 vol. % samples can be correlated to their slightly more crystalline structures after calcination (Figure 7 a). This trend is also observed for the normalised copper surface areas. This indicates that the metallic copper phase is most effectively utilised if using water contents between 10–20 vol. %. The high level of dilution and mixing between the Cu and ZnO phases facilitates and stabilises optimum nanoscale Cu^0^ crystallites.

**Figure 9:**
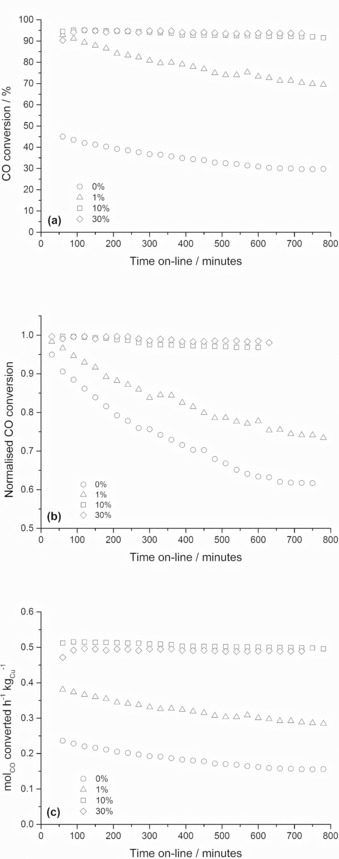
Time on‐line water‐gas‐shift testing of selected Cu/ZnO catalysts prepared by SAS precipitation using various H_2_O/EtOH solvent mixtures, with water vol. % contents indicated. (a) CO conversion, (b) CO conversion normalised to initial CO conversions and (c) catalyst activity normalised to mass of copper in final‐state catalysts.

From the extensive characterisation performed on the copper‐zinc materials, catalysts prepared using 0, 1, 10 and 30 vol. % water contents were specifically chosen for WGS testing (Figure 9). This enabled an acetate‐derived catalyst, a biphasic acetate/zincian georgeite‐derived catalyst and two zincian georgeite‐derived catalysts, with distinctly different microstructural properties, to be examined. Although testing was performed at ambient pressure, all other reaction conditions were made similar to those typical for commercial copper‐based catalysts.^1^ The two zincian georgeite derived catalysts displayed the highest WGS activity, with CO conversions of 92–93 % after 10 hours testing (Figure 9 a). These catalysts had almost identical performances despite the catalyst prepared with 30 vol. % water only having 70 % of the copper surface displayed for the 10 vol. % catalyst. In our previous work, we also determined that the WGS activity did not directly correlate with copper surface area.^30^ We found that the copper surface areas markedly decreased after reaction and concluded that specific copper–zinc interactions and low sodium impurity levels are instead essential characteristics of an optimised catalyst. The catalysts derived from acetate precursors were less active in comparison. However after the same duration, CO conversions of 30 and 66 % were still obtained despite these catalysts having negligible copper surface areas. The zincian georgeite derived catalysts also illustrated superior stability to the acetate‐derived catalysts. Activities were normalised to initial activities and showed that the zincian georgeite derived catalysts only deactivated by 3 % after 10 h testing (Figure 9 b). The catalyst stability was found to decrease with decreasing amount of zincian georgeite in the original catalyst precursor, with the catalyst prepared with 0 vol. % water deactivating by 39 %. However, we also noted that all the catalysts would have lost mass to different extents after reduction and prior to testing as a result of having different Cu/Zn molar ratios, Cu oxidation states and possibly having residual carbonate still present. Therefore, catalyst activities were normalised to copper mass (Figure 9 c). Similar trends with respect to WGS activity and stability were still apparent. Taking into account these initial and final activities, the zincian georgeite derived catalysts still only deactivated 3 % whereas the acetate‐derived catalysts deactivated between 29–34 %. All the catalysts were prepared by using the same reagents and would therefore be expected to have similar sodium content. This indicates that differences in these catalyst performances must be attributed to copper–zinc interactions and copper surface area. The choice of catalyst precursor may therefore be a key parameter in the preparation of optimal WGS catalysts, with zincian georgeite precursors displaying high activity and stability as a result of optimisation of these parameters.

## Conclusions

We have shown that the presence of water has significant implications on the physicochemical properties of copper–zinc precipitates prepared by supercritical (sc) CO_2_ antisolvent (SAS) precipitation. An amorphous bimetallic phase mixture, consisting of a highly disordered acetate and zincian georgeite, is always produced that can be fine‐tuned by varying the water/ethanol ratio. Optimum SAS precipitation occurs in the absence of water, with gas‐like mixing between EtOH and scCO_2_ resulting in intimately mixed, high‐surface‐area acetates. Unfortunately, these acetates decompose exothermically to metal oxides, which results in enhanced crystallisation, low surface areas and partial reduction. Addition of appropriate amounts of water to the SAS system results in a change in the precipitation mechanism and the formation of zincian georgeite. High water contents result in poorer catalysts and lower yields, as water and scCO_2_ are poorly miscible in the precipitation step. However, thermal treatment of zincian georgeite precursors prepared with intermediate water content, under appropriate conditions, produces highly dispersed CuO and ZnO crystallites of <5 nm in size, immersed inside a carbonate matrix. The testing of selected Cu/ZnO catalysts in the water‐gas‐shift (WGS) reaction revealed that the Cu surface area is not directly correlated to the catalyst activity. The importance of the choice of precursor was outlined. Zincian georgeite derived catalysts illustrate exceptional activity and stability in comparison with acetate‐derived catalysts, which is attributed to enhanced copper–zinc interactions in combination with a high copper surface area. It can therefore be concluded that water contents between 10–15 vol. % are optimum in the preparation of Cu/ZnO catalysts by SAS precipitation.

## Experimental Section

### Raw materials

Copper(II) acetate monohydrate (puriss. p.a., ≥99.0 %), zinc(II) acetate dihydrate (puriss. p.a., ≥99.0 %) and high‐purity atomic emission standards for copper, zinc and sodium (TraceCERT, 1000 mg L^−1^ in 2 % nitric acid) were all purchased from Sigma–Aldrich. Ethanol (absolute 99.8 %, Certified AR) was purchased from Fischer Scientific and CO_2_ (CP grade) was provided by BOC. All purchased materials were used as received. Deionised water was provided in‐house.

### SAS precipitation

The SAS‐precipitated materials were prepared as follows; Cu(OAc)_2_⋅H_2_O (4 mg mL^−1^) and Zn(OAc)_2_⋅2 H_2_O (2.13 mg mL^−1^) salts were dissolved in ethanol containing 0, 0.5, 1, 5, 10, 15, 20 and 30 % water by volume to give a nominal Cu/Zn molar ratio of 2:1 in each case. SAS precipitation experiments were performed by using apparatus manufactured by Separex. Liquefied CO_2_ was pumped at a flow rate of 6.5 kg h^−1^ and the whole system was pressurised to 110 bar and held at 40 °C. Initially, pure solvent was pumped through the fine capillary into the precipitation vessel, with a flow rate of 6.5 mL min^−1^ for 15 min, in co‐current mode with scCO_2_ to obtain steady‐state conditions inside the vessel. After this initial period, the flow of liquid solvent was stopped and the mixed acetate solution was delivered at a flow rate of 6.5 mL min^−1^. This gave a scCO_2_/metal solution molar ratio of 22:1. When all the solution had been processed, a drying step was performed. Pure ethanol was pumped at 6.5 mL min^−1^ co‐currently with scCO_2_ for 30 min, before leaving with just scCO_2_ to pump for a further hour. This was to wash the vessel in case residual solvent condensed during depressurisation and partly solubilised the precipitated powder, modifying its morphology. When the drying step was completed the scCO_2_ flow rate was stopped, the vessel was depressurised to atmospheric pressure and the precipitate was collected. Experiments were conducted for approximately 3.5 h, which resulted in the synthesis of approximately 1.1–1.5 g of solid. Recovered samples were then calcined at 300 °C in static air (ramp rate 1 °C min^−1^, 4 h).

### Characterisation

X‐ray diffraction (XRD) analysis of the materials produced was performed on a (*θ*–*θ*) PANalytical X′pert Pro powder diffractometer with a Ni filtered Cu_Kα_ radiation source operating at 40 keV and 40 mA. Patterns were recorded over the 2 *θ* angular range 10–80 ° using a step size of 0.016° with crystallite sizes determined using the Scherrer equation. Synchrotron partial distribution functions (PDF) were derived from XRD data collected on the 11‐ID‐B beamline at the Advanced Photon Source at Argonne National Laboratory, USA. Powder samples were packed into Kapton capillaries having an internal diameter of 1 mm. Room temperature powder XRD data were collected at a wavelength of 0.2114 Å using the Rapid Acquisition PDF method. The scattering data (0.5≤*Q*≤22 Å^−1^) was processed into a PDF using the program GudrunX.

FTIR spectroscopy on the materials was performed on a Shimadzu IR Affinity‐1 spectrometer operating in transmission mode over the range 400–4000 cm^−1^. Samples were pressed directly onto the sample stage without requiring the use of KBr. Alternatively, a Jasco 660 plus spectrometer was used which did require diluting samples with KBr. Raman spectroscopy was performed using a Renishaw inVia microscope with a green argon ion laser (*λ*=514 nm).

SEM and EDX analysis was performed by dispersing catalysts on adhesive carbon discs, mounted on 12.5 mm aluminium stubs. Analysis was performed using a Carl Zeiss Evo 40 microscope, operated at 5–20 kV and 50–2000 pA. Microwave plasma atomic emission spectroscopy (MP‐AES) was performed by using a 4100 MP‐AES manufactured by Agilent Technologies. Solid samples and effluents were digested in 20 vol. % HNO_3_/H_2_O solutions and compositions quantified against calibration standards.

TGA and DTA were performed using a Setaram Labsys 1600 instrument. Samples (5–30 mg) were loaded into alumina crucibles and heated to 600 °C at 5 °C min^−1^ in a flow of synthetic air (50 mL min^−1^). For all TGA runs, blank runs were subtracted from the relevant data to remove buoyancy effects.

BET surface‐area analysis was performed using a Micromeritics Gemini 2360 surface analyser. Isotherms were obtained using N_2_ at −196.15 °C with surface area analysis performed using a 5 point BET plot in the *P*/*P*_0_ range 0.06–0.35. Cu surface‐area analysis was performed on a Quantachrome ChemBET chemisorption analyser equipped with a thermal‐conductivity detector (TCD). Calcined samples (100 mg) were reduced to catalysts using 10 % H_2_/Ar (30 mL min^−1^) with heating to 140 °C at 10 °C min^−1^, and then to 225 °C at 1 °C min^−1^. The resulting catalysts were cooled to 65 °C under He for N_2_O pulsing. 12 N_2_O pulses (113 μl each) were followed with 3 N_2_ pulses for calibration. The amount of N_2_ emitted was assumed to amount to half a monolayer coverage of oxygen and that the surface density of Cu is 1.47×10^19^ atoms m^−2^.

### WGS testing

Water‐gas‐shift testing was performed on a custom‐made fixed‐bed flow reactor. The catalyst (0.1 g), in powder form, was packed into a stainless‐steel tube using quartz wool and a mesh to hold the catalyst in place within the bed. Catalysts were reduced by using a 1 % H_2_/N_2_ feed whilst heating to 130 °C at 5 °C min^−1^ and then to 225 °C at 1 °C min^−1^. The catalysts were held at 225 °C for an hour to ensure complete reduction had taken place. The gas feed was then switched to 1.1 % CO, 4.3 % CO_2_, 24.0 % H_2_O, 13.8 % H_2_ with N_2_ as balance. The gases were introduced to the catalyst bed using mass flow controllers (Bronkhorst). The water was passed through a liquid‐flow controller (Bronkhorst) into a controlled evaporator mixer heated to 140 °C where it was mixed with the carrier gas, N_2_. The total flow rate was 50 mL min^−1^ with the reactions performed at ambient pressure. Products were quantified using an on‐line Gasmet Dx4000 Fourier Transform infrared spectrometer where a spectrum was recorded every minute.

Information on the data underpinning the results presented here, including how to access them, can be found at Cardiff University data catalogue at doi.org/10.17035/d.2017.0033156623

## Conflict of interest

*The authors declare no conflict of interest*.

## Supporting information

As a service to our authors and readers, this journal provides supporting information supplied by the authors. Such materials are peer reviewed and may be re‐organized for online delivery, but are not copy‐edited or typeset. Technical support issues arising from supporting information (other than missing files) should be addressed to the authors.

Supplementary
