# Supplementary material for: Supercritical Antisolvent Precipitation of Amorphous Copper–Zinc Georgeite and Acetate Precursors for the Preparation of Ambient‐Pressure Water‐Gas‐Shift Copper/Zinc Oxide Catalysts
Source: ChemCatChem. 2017 Apr 7;9(9):1621–31. doi: 10.1002/cctc.201601603 (PMC5485020; doi:10.1002/cctc.201601603)
Supplement: Supplementary file 1 — Supplementary [file CCTC-9-1621-s001.pdf]

Heterogeneous & Homogeneous & Bio- & Nano-
CHEMCATCHEM
CATALYSIS

Supporting Information

Supercritical Antisolvent Precipitation of Amorphous Copper–Zinc Georgeite and Acetate Precursors for the Preparation of Ambient-Pressure Water-Gas-Shift Copper/Zinc Oxide Catalysts

Paul J. Smith,[a] Simon A. Kondrat,[a] James H. Carter,[a] Philip A. Chater,[b] Jonathan K. Bartley,[a] Stuart H. Taylor,[a] Michael S. Spencer,[a] and Graham J. Hutchings*[a]

cctc_201601603_sm_miscellaneous_information.pdf

Supporting information

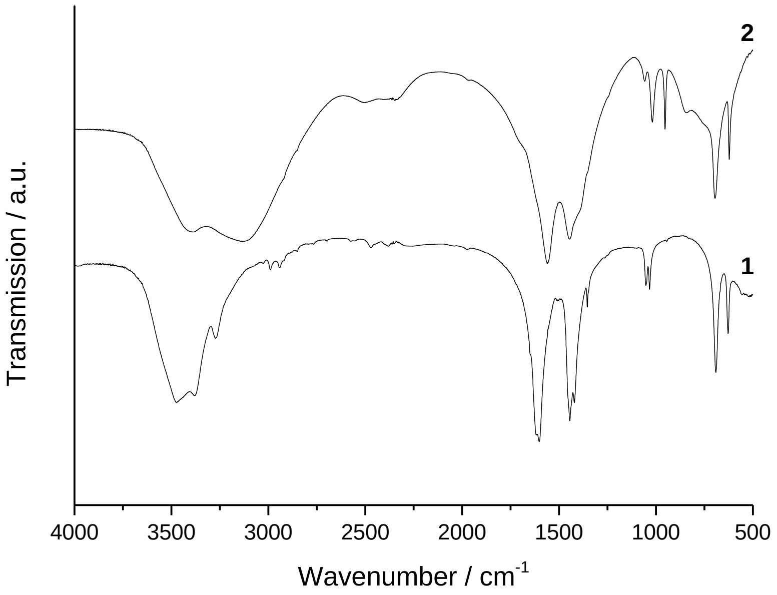

Figure S1. FT-IR spectra of metal acetate starting materials: (1) copper (II) acetate monohydrate and (2) zinc (II) acetate dihydrate.

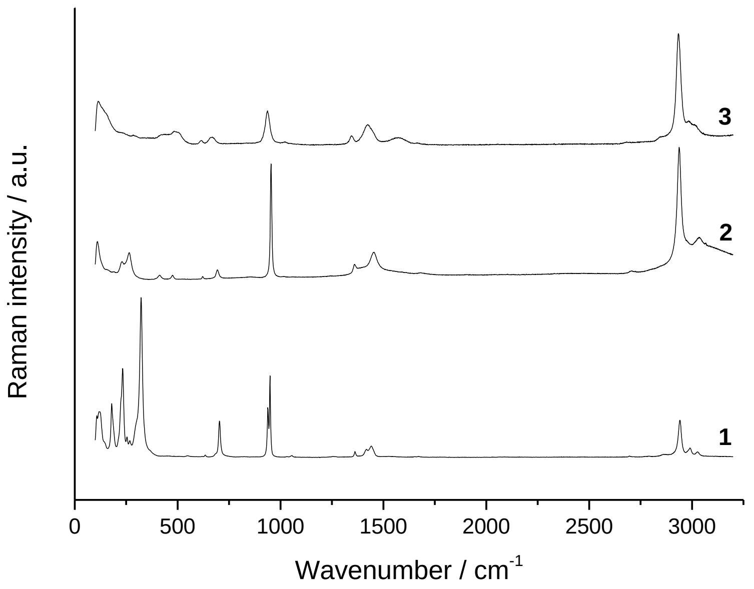

Figure S2. Raman spectra of metal acetate materials: (1) copper (II) acetate monohydrate (2) zinc (II) acetate dihydrate and (3) precipitated copper-zinc acetate obtained from starting metal acetate solution.

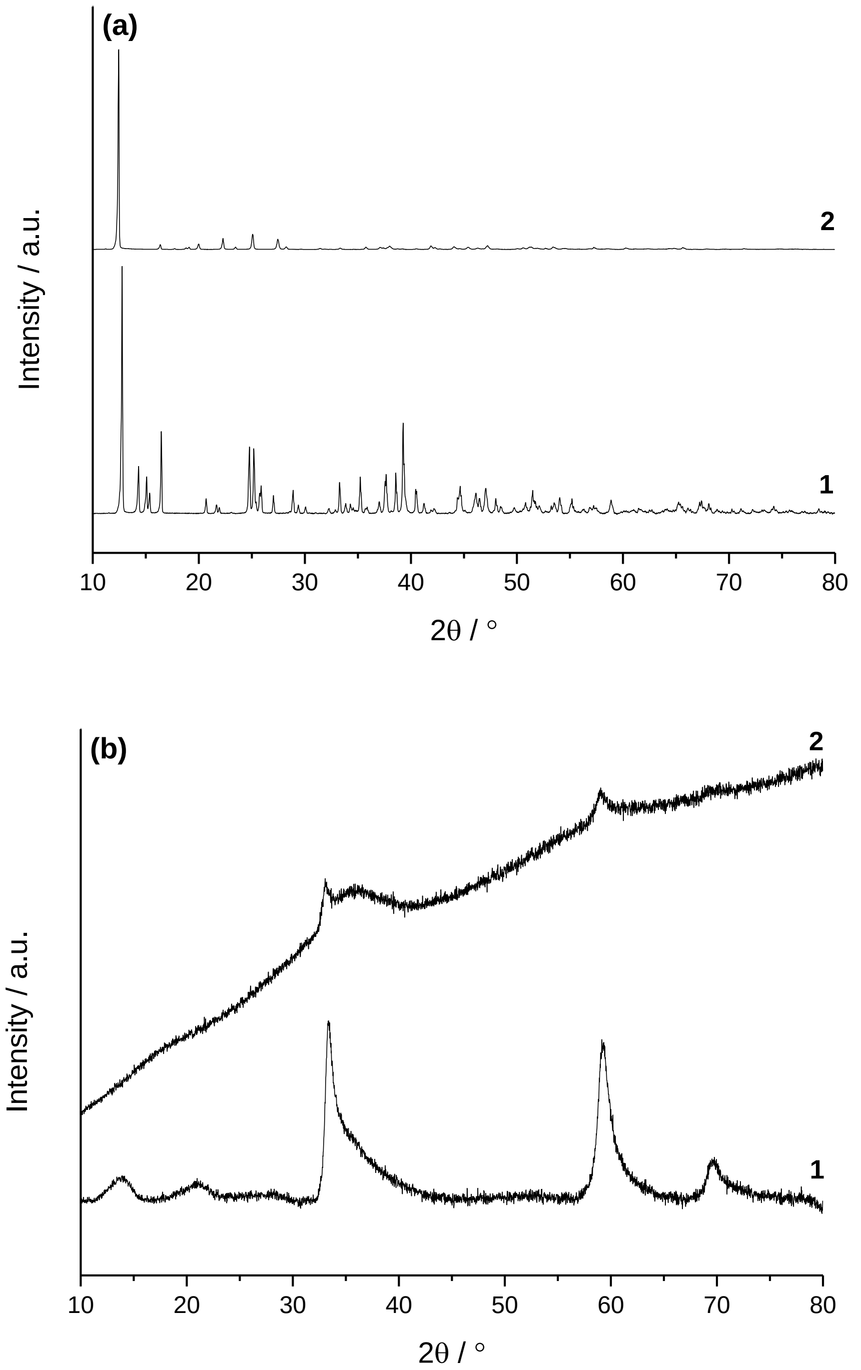

Figure S3. XRD patterns of (a) as-received acetate materials: (1) copper (II) acetate monohydrate and (2) zinc (II) acetate dehydrate. (b) Comparison on XRD patterns of (1) copper-zinc acetate precipitate obtained from starting metal acetate solution and (2) zincian georgeite prepared with 10 vol. % H_2O/EtOH. Reflections at 33.4 and 59.1 ° found in zincian georgeite samples clearly match with acetate precipitate.
